# Supplementary material for: State-space kinetic Ising model reveals task-dependent entropy flow in sparsely active nonequilibrium neuronal dynamics
Source: Nat Commun. 2025 Dec 9;16:10852. doi: 10.1038/s41467-025-66669-w (PMC12689823; doi:10.1038/s41467-025-66669-w)
Supplement: Supplementary file 1 — Supplementary Information [file 41467_2025_66669_MOESM1_ESM.pdf]

State-space kinetic Ising model reveals task-dependent entropy flow in sparsely active nonequilibrium neuronal dynamics

Supplementary Information

Ken Ishihara
Graduate School of Life Sciences, Hokkaido University, Sapporo, Japan
Center for Human Nature, Artificial Intelligence,
and Neuroscience (CHAIN), Hokkaido University, Sapporo, Japan

Hideaki Shimazaki
Graduate School of Informatics, Kyoto University, Kyoto, Japan
Center for Human Nature, Artificial Intelligence,
and Neuroscience (CHAIN), Hokkaido University, Sapporo, Japan

Supplementary Note 1: State-space kinetic Ising model

In this Supplementary Note, we provide the filtering and smoothing algorithms for the time-varying kinetic Ising model and an optimization method of its hyperparameters via the Expectation-Maximization algorithm.

1. Model

Let $x_{i,t} = \{0, 1\}$ be an outcome of a binary random variable of neuron i at time t ($i = 1, \ldots, N$, $t = 0, \ldots, T$). In the kinetic Ising model, the activation of neuron i at time t independently depends on the activities of the neurons in the previous time step $t-1$. The conditional probability mass function of $x_{i,t}$ is given as

$$p(x_{i,t}|x_{1,t-1}, \ldots, x_{N,t-1}, \boldsymbol{\theta}_t^i) = \frac{\exp\left[\theta_{i,t}x_{i,t} + \sum_{j=1}^N \theta_{ij,t}x_{i,t}x_{j,t-1}\right]}{1 + \exp\left[\theta_{i,t} + \sum_{j=1}^N \theta_{ij,t}x_{j,t-1}\right]}, \tag{S1.1}$$

where $\theta_{i,t}$ is a time-dependent field parameter that determines the bias for inputs to the i-th neuron at time t, and $\theta_{ij,t}$ is a time-dependent coupling parameter from the j-th neuron to the i-th neuron at time t. These parameters are collectively denoted as $\boldsymbol{\theta}_t^i = (\theta_{i,t}, \theta_{i1,t}, \ldots \theta_{ij,t}, \ldots \theta_{iN,t})$. Using the log normalization function,

$$\psi(\boldsymbol{\theta}_t^i, \mathbf{x}_{t-1}^l) = \log\left[1 + \exp\left[\theta_{i,t} + \sum_{j=1}^N \theta_{ij,t}x_{j,t-1}^l\right]\right], \tag{S1.2}$$

the kinetic Ising model is also written as

$$p(x_{i,t}|x_{1,t-1}, \ldots, x_{N,t-1}, \boldsymbol{\theta}_t^i) = \exp\left[\theta_{i,t}x_{i,t} + \sum_{j=1}^N \theta_{ij,t}x_{i,t}x_{j,t-1} - \psi(\boldsymbol{\theta}_t^i, \mathbf{x}_{t-1})\right]. \tag{S1.3}$$

Assuming conditional independence, the joint probability mass function that determines the probabilities of generating patterns of activity across N neurons is given by

$$\prod_{i=1}^N p(x_{i,t}|x_{1,t-1}, \ldots, x_{N,t-1}, \boldsymbol{\theta}_t^i). \tag{S1.4}$$

Typical neurophysiological experiments repeat multiple trials of measurement under the same experimental conditions. We let $x_{i,t}^l = \{0, 1\}$ be a binary variable of the i-th neuron at time t in the l-th trial ($i = 1, \ldots, N$, $t = 0, \ldots, T$, $l = 1, \ldots, L$). We collectively denote the binary patterns of simultaneously recorded neurons at time t in the l-th trial using a vector, $\mathbf{x}_t^l = (x_{1,t}^l, \ldots, x_{N,t}^l)$. Further, we denote the patterns at time t from all trials by $\mathbf{x}_t = (\mathbf{x}_t^1, \ldots, \mathbf{x}_t^l, \ldots, \mathbf{x}_t^L)$ and denote all the patterns up to time t by $\mathbf{x}_{0:t}$. We use the same convention for the time-varying parameters, denoting them as $\boldsymbol{\theta}_t = (\boldsymbol{\theta}_t^1, \ldots, \boldsymbol{\theta}_t^i, \ldots, \boldsymbol{\theta}_t^N)$ and $\boldsymbol{\theta}_{1:t}$ for their trajectories over time.

Given the time-varying parameters $\boldsymbol{\theta}_{1:T}$, the probability mass function observing binary sequences $\mathbf{x}_{0:T}$ is given as

$$p(\mathbf{x}_{0:T}|\boldsymbol{\theta}_{1:T}) = \prod_{l=1}^{L}\prod_{i=1}^{N}\left[p(x_{i,0}^{l})\prod_{t=1}^{T}p(x_{i,t}^{l}|\mathbf{x}_{t-1}^{l},\boldsymbol{\theta}_{t}^{i})\right], \tag{S1.5}$$

where we use $p(x_{i,0}^{l}) = 0.5$ for data generation. We assume that the same time-dependent parameters apply across trials.

In the state-space model, the state model defines the discrete-time stochastic processes of the latent variables, which are the time-varying parameters $\boldsymbol{\theta}_{0:T}$ in our model. We use the following Gaussian model by assuming independent processes across neurons:

$$p(\boldsymbol{\theta}_{0:T}) = \prod_{i=1}^{N}\left[p(\boldsymbol{\theta}_{0}^{i}|\boldsymbol{\mu}^{i},\boldsymbol{\Sigma}^{i})\prod_{t=1}^{T}p(\boldsymbol{\theta}_{t}^{i}|\boldsymbol{\theta}_{t-1}^{i},Q^{i})\right], \tag{S1.6}$$

where the transition of the i-th neuron is given by

$$p(\boldsymbol{\theta}_{t}^{i}|\boldsymbol{\theta}_{t-1}^{i},\mathbf{Q}^{i}) = \frac{1}{\sqrt{|2\pi\mathbf{Q}^{i}|}}\exp\left[-\frac{1}{2}(\boldsymbol{\theta}_{t}^{i}-\boldsymbol{\theta}_{t-1}^{i})^{\top}(\mathbf{Q}^{i})^{-1}(\boldsymbol{\theta}_{t}^{i}-\boldsymbol{\theta}_{t-1}^{i})\right] \tag{S1.7}$$

with $\mathbf{Q}^{i}$ being the noise covariance for the transition of the i-th neuron. The initial density of the i-th neuron $p(\boldsymbol{\theta}_{0}^{i}|\boldsymbol{\mu}^{i},\boldsymbol{\Sigma}^{i})$ is given as a Gaussian distribution with mean $\boldsymbol{\mu}^{i}$ and covariance $\boldsymbol{\Sigma}^{i}$. In practice, we used a zero vector and a unit matrix before optimization, respectively. In the followings, we denote a set of hyperparameters $\boldsymbol{\mu}^{i},\boldsymbol{\Sigma}^{i},\mathbf{Q}^{i}$ for $i=1,\ldots,N$ collectively by $\mathbf{w}$.

2. One-step prediction density

In this section, we derive the one-step prediction density $p(\boldsymbol{\theta}_{t}|\mathbf{x}_{0:t-1},\mathbf{w})$, using Chapman–Kolmogorov's equation.

For $t=1$, we note that the one-step prediction is specified as a prior distribution: $p(\boldsymbol{\theta}_{1}|\mathbf{x}_{0},\mathbf{w}) = p(\boldsymbol{\theta}_{1}|\mathbf{w}) = \prod_{i=1}^{N}\mathcal{N}(\boldsymbol{\theta}_{1}^{i};\boldsymbol{\mu}^{i},\boldsymbol{\Sigma}^{i})$. For $t=2,\ldots,T$, the one-step prediction density is computed via the Chapman–Kolmogorov equation:

$$\begin{aligned} p(\boldsymbol{\theta}_{t}|\mathbf{x}_{0:t-1},\mathbf{w}) &= \int p(\boldsymbol{\theta}_{t},\boldsymbol{\theta}_{t-1}|\mathbf{x}_{0:t-1},\mathbf{w})\,d\boldsymbol{\theta}_{t-1} \\ &= \int p(\boldsymbol{\theta}_{t}|\boldsymbol{\theta}_{t-1},\mathbf{x}_{0:t-1},\mathbf{w})\,p(\boldsymbol{\theta}_{t-1}|\mathbf{x}_{0:t-1},\mathbf{w})\,d\boldsymbol{\theta}_{t-1}, \end{aligned} \tag{S1.8}$$

where $p(\boldsymbol{\theta}_{t-1}|\mathbf{x}_{0:t-1},\mathbf{w})$ is the filter density at time $t-1$. We assume that the filter density factors into a product of individual neurons. Coupled with the factorized assumption of the state model, this leads to the factorization of the one-step prediction density:

$$p(\boldsymbol{\theta}_{t}|\mathbf{x}_{0:t-1},\mathbf{w}) = \prod_{i=1}^{N}\int p(\boldsymbol{\theta}_{t}^{i}|\boldsymbol{\theta}_{t-1}^{i},\mathbf{w})\,p(\boldsymbol{\theta}_{t-1}^{i}|\mathbf{x}_{0:t-1},\mathbf{w})\,d\boldsymbol{\theta}_{t-1}^{i}. \tag{S1.9}$$

We further assume that the filter density at time $t-1$, $p(\boldsymbol{\theta}_{t-1}^{i}|\mathbf{x}_{0:t-1},\mathbf{w})$, is approximated by a Gaussian distribution with mean $\boldsymbol{\theta}_{t-1|t-1}^{i}$ and covariance $\mathbf{W}_{t-1|t-1}^{i}$ (to be justified at the next filtering step):

$$p(\boldsymbol{\theta}_{t-1}^{i}|\mathbf{x}_{0:t-1},\mathbf{w}) = \mathcal{N}(\boldsymbol{\theta}_{t-1}^{i};\boldsymbol{\theta}_{t-1|t-1}^{i},\mathbf{W}_{t-1|t-1}^{i}). \tag{S1.10}$$

Here the filter mean is defined as

$$\boldsymbol{\theta}_{t-1|t-1}^{i} = \int p(\boldsymbol{\theta}_{t-1}^{i}|\mathbf{x}_{0:t-1})\boldsymbol{\theta}_{t-1}^{i}d\boldsymbol{\theta}_{t-1}^{i} = E_{\boldsymbol{\theta}_{t-1}^{i}|\mathbf{x}_{0:t-1}}\boldsymbol{\theta}_{t-1}^{i}. \tag{S1.11}$$

It represents the expected value of the parameter at time $t-1$ using data up to $t-1$. The filter covariance is

$$\mathbf{W}_{t-1|t-1}^{i} = E_{\boldsymbol{\theta}_{t-1}^{i}|\mathbf{x}_{0:t-1}}(\boldsymbol{\theta}_{t-1}^{i}-E_{\boldsymbol{\theta}_{t-1}^{i}|\mathbf{x}_{0:t-1}}\boldsymbol{\theta}_{t-1}^{i})(\boldsymbol{\theta}_{t-1}^{i}-E_{\boldsymbol{\theta}_{t-1}^{i}|\mathbf{x}_{0:t-1}}\boldsymbol{\theta}_{t-1}^{i})^{\top}. \tag{S1.12}$$

Given the Gaussian transition model $p(\boldsymbol{\theta}_t^i|\boldsymbol{\theta}_{t-1}^i, \mathbf{w}) = \mathcal{N}(\boldsymbol{\theta}_t^i; \boldsymbol{\theta}_{t-1}^i, \mathbf{Q}^i)$, the one-step prediction density $p(\boldsymbol{\theta}_t|\mathbf{x}_{0:t-1}, \mathbf{w})$ becomes a Gaussian distribution. Namely, by completing the square with resect to $\boldsymbol{\theta}_t^i$ and calculating the integral, we obtain

$$p(\boldsymbol{\theta}_t|\mathbf{x}_{0:t-1}, \mathbf{w}) = \prod_{i=1}^{N} \mathcal{N}\big(\boldsymbol{\theta}_t; \boldsymbol{\theta}_{t|t-1}^i,\ \mathbf{W}_{t|t-1}^i\big), \tag{S1.13}$$

where

$$\boldsymbol{\theta}_{t|t-1}^i = \boldsymbol{\theta}_{t-1|t-1}^i, \tag{S1.14}$$

$$\mathbf{W}_{t|t-1}^i = \mathbf{W}_{t-1|t-1}^i + \mathbf{Q}^i. \tag{S1.15}$$

We also define $\boldsymbol{\theta}_{1|0}^i = \boldsymbol{\mu}^i$ and $\mathbf{W}_{t|0}^i = \boldsymbol{\Sigma}^i$ for the consistent notation of the one-step prediction density for $t = 1, \ldots, T$ in subsequent calculations.

3. Filtering

Using the observation model and the one-step prediction density $p(\boldsymbol{\theta}_t|\mathbf{x}_{0:t-1}, \mathbf{w})$, the posterior filter density is given as

$$p(\boldsymbol{\theta}_t|\mathbf{x}_{0:t}, \mathbf{w}) \propto \prod_{i=1}^{N}\prod_{l=1}^{L} \exp\left[\theta_{i,t}x_{i,t}^l + \sum_{j=1}^{N}\theta_{ij,t}x_{it}^l x_{j,t-1}^l - \psi(\boldsymbol{\theta}_t^i, \mathbf{x}_{t-1}^l)\right]$$
$$\cdot \prod_{i=1}^{N} \exp\left[-\frac{1}{2}(\boldsymbol{\theta}_t^i - \boldsymbol{\theta}_{t|t-1}^i)^\top (\mathbf{W}_{t|t-1}^i)^{-1}(\boldsymbol{\theta}_t^i - \boldsymbol{\theta}_{t|t-1}^i)\right]. \tag{S1.16}$$

This expression confirms that the filter density at time t is a product of the individual neurons' filter densities, validating the assumption of independent filter densities in constructing the one-step prediction density. The result enables independent filtering for each neuron.

We now approximate the filter density by the Gaussian distribution using Laplace's method. Namely, we obtain the maximum a posteriori (MAP) estimate of the filter density and use the Hessian at around the MAP estimate to obtain the approximate covariance. Using

$$\boldsymbol{\theta}_t^i = [\theta_{i,t}, \theta_{i1,t}, ...\theta_{iN,t}]^\top, \tag{S1.17}$$

$$\mathbf{F}(x_{i,t}^l, \mathbf{x}_{t-1}^l) = \left[x_{i,t}^l,\ x_{i,t}^l x_{1,t-1}^l,\ x_{i,t}^l x_{2,t-1}^l,\ \ldots,\ x_{i,t}^l x_{N,t-1}^l\right]^\top, \tag{S1.18}$$

we have

$$p(\boldsymbol{\theta}_t|\mathbf{x}_{0:t}, \mathbf{w}) \propto \prod_{i=1}^{N} \exp\left[\sum_{l=1}^{L}(\boldsymbol{\theta}_t^i)^\top \mathbf{F}(x_{i,t}^l, \mathbf{x}_{t-1}^l) - \psi(\boldsymbol{\theta}_t^i, \mathbf{x}_{t-1}^l)\right.$$
$$\left. - \frac{1}{2}(\boldsymbol{\theta}_t^i - \boldsymbol{\theta}_{t|t-1}^i)^\top (\mathbf{W}_{t|t-1}^i)^{-1}(\boldsymbol{\theta}_t^i - \boldsymbol{\theta}_{t|t-1}^i)\right], \tag{S1.19}$$

where $\psi(\boldsymbol{\theta}_t^i, \mathbf{x}_{t-1}^l)$ is now given as

$$\psi(\boldsymbol{\theta}_t^i, \mathbf{x}_{t-1}^l) = \log\left[1 + \exp\left[(\boldsymbol{\theta}_t^i)^\top \mathbf{F}(1, \mathbf{x}_{t-1}^l)\right]\right]. \tag{S1.20}$$

First, we obtain the MAP estimate defined as

$$\boldsymbol{\theta}_{\mathrm{MAP}} = \arg\max_{\boldsymbol{\theta}_t} \log p(\boldsymbol{\theta}_t|\mathbf{x}_{0:t}, \mathbf{w}). \tag{S1.21}$$

We obtain the MAP estimate through numerical optimization using the Newton–Raphson method. Notably, the MAP estimate for each neurons, $\boldsymbol{\theta}_{\mathrm{MAP}}^i$, can be obtained independently of the others. For this goal, we obtain the first and second-order derivatives of the log posterior with respect to $\boldsymbol{\theta}_t^i$. The first-order derivative with respect to $\boldsymbol{\theta}_t^i$ results in

$$\frac{\partial \log p(\boldsymbol{\theta}_t|\mathbf{x}_{0:t}, \mathbf{w})}{\partial \boldsymbol{\theta}_t^i} = \sum_{l=1}^{L}\left[\mathbf{F}(x_{i,t}^l, \mathbf{x}_{t-1}^l) - \frac{\partial \psi(\boldsymbol{\theta}_t^i, \mathbf{x}_{t-1}^l)}{\partial \boldsymbol{\theta}_t^i}\right] - (\mathbf{W}_{t|t-1}^i)^{-1}(\boldsymbol{\theta}_t^i - \boldsymbol{\theta}_{t|t-1}^i). \tag{S1.22}$$

Here, the derivative of $\psi(\boldsymbol{\theta}_t^i, \mathbf{x}_{t-1}^l)$ with respect to $\boldsymbol{\theta}_t^i$ is given by:

$$\begin{aligned}\frac{\partial\psi(\boldsymbol{\theta}_t^i, \mathbf{x}_{t-1}^l)}{\partial\boldsymbol{\theta}_t^i} &= \frac{\exp\left[(\boldsymbol{\theta}_t^i)^\top \mathbf{F}(1, \mathbf{x}_{t-1}^l)\right]}{1+\exp\left[(\boldsymbol{\theta}_t^i)^\top \mathbf{F}(1, \mathbf{x}_{t-1}^l)\right]}\mathbf{F}(1, \mathbf{x}_{t-1}^l) \\ &= \exp\left[(\boldsymbol{\theta}_t^i)^\top \mathbf{F}(1, \mathbf{x}_{t-1}^l) - \psi(\boldsymbol{\theta}_t^i, \mathbf{x}_{t-1}^l)\right]\mathbf{F}(1, \mathbf{x}_{t-1}^l) \\ &= r_{i,t}^l(\mathbf{x}_{t-1}^l)\mathbf{F}(1, \mathbf{x}_{t-1}^l),\end{aligned} \tag{S1.23}$$

where we defined the expected rate of i-th neuron at time t given the activity of the previous time step $\mathbf{x}_{t-1}^l$ as

$$\begin{aligned}r_{i,t}^l(\mathbf{x}_{t-1}^l) &\equiv E_{x_{i,t}^l|\mathbf{x}_{t-1}^l} x_{i,t}^l \\ &= \sum_{x_{i,t}^l} p(x_{i,t}^l|\mathbf{x}_{t-1}^l)\, x_{i,t}^l \\ &= \exp\left[(\boldsymbol{\theta}_t^i)^\top \mathbf{F}(1, \mathbf{x}_{t-1}^l) - \psi(\boldsymbol{\theta}_t^i, \mathbf{x}_{t-1}^l)\right].\end{aligned} \tag{S1.24}$$

The second derivative of $\log p(\boldsymbol{\theta}_t|\mathbf{x}_{0:t}, \mathbf{w})$ with respect to $\boldsymbol{\theta}_t^i$ is given by

$$\frac{\partial}{\partial\boldsymbol{\theta}_t^i}\left(\frac{\partial \log p(\boldsymbol{\theta}_t|\mathbf{x}_{0:t}, \mathbf{w})}{\partial(\boldsymbol{\theta}_t^i)^\top}\right) = \sum_{l=1}^{L}\left[-\frac{\partial^2\psi(\boldsymbol{\theta}_t^i, \mathbf{x}_{t-1}^l)}{\partial\boldsymbol{\theta}_t^i\partial(\boldsymbol{\theta}_t^i)^\top}\right] - (\mathbf{W}_{t|t-1}^i)^{-1}. \tag{S1.25}$$

The second derivative of $\psi(\boldsymbol{\theta}_t^i, \mathbf{x}_{t-1}^l)$ with respect to $\boldsymbol{\theta}_t^i$ is given by:

$$\begin{aligned}\frac{\partial^2\psi(\boldsymbol{\theta}_t^i, \mathbf{x}_{t-1}^l)}{\partial\boldsymbol{\theta}_t^i(\boldsymbol{\theta}_t^i)^\top} &= \frac{\partial}{\partial\boldsymbol{\theta}_t^i}\exp\left[(\boldsymbol{\theta}_t^i)^\top \mathbf{F}(1, \mathbf{x}_{t-1}^l) - \psi(\boldsymbol{\theta}_t^i, \mathbf{x}_{t-1}^l)\right]\mathbf{F}(1, \mathbf{x}_{t-1}^l)^\top \\ &= \exp\left[(\boldsymbol{\theta}_t^i)^\top \mathbf{F}(1, \mathbf{x}_{t-1}^l) - \psi(\boldsymbol{\theta}_t^i, \mathbf{x}_{t-1}^l)\right]\left[\mathbf{F}(1, \mathbf{x}_{t-1}^l) - \frac{\partial\psi(\boldsymbol{\theta}_t^i, \mathbf{x}_{t-1}^l)}{\partial\boldsymbol{\theta}_t^i}\right]\mathbf{F}(1, \mathbf{x}_{t-1}^l)^\top \\ &= r_{i,t}^l(\mathbf{x}_{t-1}^l)\{1 - r_{i,t}^l(\mathbf{x}_{t-1}^l)\}\mathbf{F}(1, \mathbf{x}_{t-1}^l)\mathbf{F}(1, \mathbf{x}_{t-1}^l)^\top.\end{aligned} \tag{S1.26}$$

Using the first and second-order derivatives, the MAP estimate $\boldsymbol{\theta}_{\mathrm{MAP}}^i$ for each neurons was found by the Newton-Raphson method.

After finding the MAP estimate, we approximate the filter density by a Gaussian distribution via the Laplace's method,

$$p(\boldsymbol{\theta}_t^i|\mathbf{x}_{1:t}, \mathbf{w}) = \frac{1}{\sqrt{|2\pi\mathbf{W}_{t|t}|}}\exp\left[-\frac{1}{2}(\boldsymbol{\theta}_t^i - \boldsymbol{\theta}_{t|t}^i)^\top \mathbf{W}_{t|t}^{-1}(\boldsymbol{\theta}_t^i - \boldsymbol{\theta}_{t|t}^i)\right] \tag{S1.27}$$

with the following mean and variance:

$$\boldsymbol{\theta}_{t|t}^i = \boldsymbol{\theta}_{\mathrm{MAP}}^i, \tag{S1.28}$$

and

$$\begin{aligned}\mathbf{W}_{t|t}^i &= \left[-\frac{\partial}{\partial\boldsymbol{\theta}_t^i}\left(\frac{\partial \log p\left(\boldsymbol{\theta}_{t-1}^i|\mathbf{x}_{1-t}, \mathbf{w}\right)}{\partial\left(\boldsymbol{\theta}_t^i\right)^\intercal}\right)\Bigg|_{\boldsymbol{\theta}_t^i=\boldsymbol{\theta}_{t|t}^i}\right]^{-1} \\ &= \left[\mathbf{G}\left(\boldsymbol{\theta}_{t|t}^i\right) + \left(\mathbf{W}_{t|t-1}^i\right)^{-1}\right]^{-1},\end{aligned} \tag{S1.29}$$

where $\mathbf{G}\left(\boldsymbol{\theta}_t^i\right)$ is given by

$$\begin{aligned}\mathbf{G}\left(\boldsymbol{\theta}_t^i\right) &= \sum_{l=1}^{L}\frac{\partial^2\psi(\boldsymbol{\theta}_t^i, \mathbf{x}_{t-1}^l)}{\partial\boldsymbol{\theta}_t^i\partial(\boldsymbol{\theta}_t^i)^\top} \\ &= \sum_{l=1}^{L} r_{i,t}^l(\mathbf{x}_{t-1}^l)\{1 - r_{i,t}^l(\mathbf{x}_{t-1}^l)\}\mathbf{F}(1, \mathbf{x}_{t-1}^l)\mathbf{F}(1, \mathbf{x}_{t-1}^l)^\top.\end{aligned} \tag{S1.30}$$

By sequentially applying the one-step prediction density and the filter density for $t = 1, \ldots, T$, we obtain the filter densities of all time steps.

4. Smoothing

Given that the filter density is approximated by Gaussian distributions, the smoothing density for the parameters of each neuron can be computed iteratively by using the filter density and the one-step prediction density in a backward manner from the final time step T, following the Rauch-Tung-Striebel smoother [1]:

$$\boldsymbol{\theta}^i_{t-1|T} = \boldsymbol{\theta}^i_{t-1|t-1} + \mathbf{A}^i_{t-1}\left(\boldsymbol{\theta}^i_{t|T} - \boldsymbol{\theta}^i_{t|t}\right), \tag{S1.31}$$

$$\mathbf{W}^i_{t-1|T} = \mathbf{W}^i_{t-1|t-1} + \mathbf{A}^i_{t-1}\left(\mathbf{W}^i_{t|T} - \mathbf{W}^i_{t|t-1}\right)\left(\mathbf{A}^i_{t-1}\right)^\top, \tag{S1.32}$$

$$\mathbf{A}^i_{t-1} = \mathbf{W}^i_{t-1|t-1}\left(\mathbf{W}^i_{t|t-1}\right)^{-1}, \tag{S1.33}$$

for $t = 2, \dots, T$. For completeness, we provide a compact derivation of these equations below.

At the smoothing, we estimate the latent state $\boldsymbol{\theta}^i_t$ given the entire observed data $\mathbf{x}_{0:T}$. The smoother posterior density is given as

$$\begin{aligned} p(\boldsymbol{\theta}^i_{t-1}|\mathbf{x}_{0:T},\mathbf{w}) &= \int p(\boldsymbol{\theta}^i_{t-1}|\boldsymbol{\theta}^i_t,\mathbf{x}_{0:T},\mathbf{w})p(\boldsymbol{\theta}^i_t|\mathbf{x}_{0:T},\mathbf{w})\,d\boldsymbol{\theta}^i_t \\ &= \int p(\boldsymbol{\theta}^i_{t-1}|\boldsymbol{\theta}^i_t,\mathbf{x}_{0:t-1},\mathbf{w})p(\boldsymbol{\theta}^i_t|\mathbf{x}_{0:T},\mathbf{w})\,d\boldsymbol{\theta}^i_t. \end{aligned} \tag{S1.34}$$

Here, we used the Markovian assumption at the second equality. The conditional density $p(\boldsymbol{\theta}^i_{t-1}|\boldsymbol{\theta}^i_t,\mathbf{x}_{0:t-1},\mathbf{w})$ is obtained as

$$\begin{aligned} p(\boldsymbol{\theta}^i_{t-1}|\boldsymbol{\theta}^i_t,\mathbf{x}_{0:t-1},\mathbf{w}) &= \frac{p(\boldsymbol{\theta}^i_{t-1},\boldsymbol{\theta}^i_t|\mathbf{x}_{0:t-1},\mathbf{w})}{p(\boldsymbol{\theta}^i_t|\mathbf{x}_{0:t-1},\mathbf{w})} \\ &= \frac{p(\boldsymbol{\theta}^i_t|\boldsymbol{\theta}^i_{t-1},\mathbf{w})p(\boldsymbol{\theta}^i_{t-1}|\mathbf{x}_{0:t-1},\mathbf{w})}{p(\boldsymbol{\theta}^i_t|\mathbf{x}_{0:t-1},\mathbf{w})}, \end{aligned} \tag{S1.35}$$

which is composed of the filter and one-step prediction densities, and the state model. Since we assume that these are Gaussian distributions, given that the smoother density at time t is Gaussian, the linear operations in Eqs. S1.34 and S1.35 guarantee that the smoother density at time $t-1$ is Gaussian. Therefore, the distribution is specified by the mean and covariance defined as

$$\boldsymbol{\theta}^i_{t-1|T} \equiv E_{\boldsymbol{\theta}^i_{t-1}|\mathbf{x}_{0:T}}\boldsymbol{\theta}^i_{t-1} \tag{S1.36}$$

$$\mathbf{W}^i_{t-1|T} \equiv E_{\boldsymbol{\theta}^i_{t-1}|\mathbf{x}_{0:T}}\left(\boldsymbol{\theta}^i_{t-1} - \boldsymbol{\theta}^i_{t-1|T}\right)\left(\boldsymbol{\theta}^i_{t-1} - \boldsymbol{\theta}^i_{t-1|T}\right)^\top. \tag{S1.37}$$

To obtain their closed form expressions, first we note that the joint density in Eq. S1.35 is written as

$$p(\boldsymbol{\theta}^i_{t-1},\boldsymbol{\theta}^i_t|\mathbf{x}_{0:t-1},\mathbf{w}) = \mathcal{N}\left(\begin{pmatrix}\boldsymbol{\theta}^i_{t-1}\\ \boldsymbol{\theta}^i_t\end{pmatrix};\begin{pmatrix}\boldsymbol{\theta}^i_{t-1|t-1}\\ \boldsymbol{\theta}^i_{t|t-1}\end{pmatrix},\begin{pmatrix}\mathbf{W}^i_{t-1|t-1} & \mathbf{W}^i_{t-1,t|t-1}\\ \mathbf{W}^i_{t,t-1|t-1} & \mathbf{W}^i_{t|t-1}\end{pmatrix}\right), \tag{S1.38}$$

where $\mathbf{W}^i_{t-1,t|t-1}$ is the cross covariance given the data up to time $t-1$. Here, we note that, under the linear Gaussian transition with an identity transition matrix, the one-step prediction mean is

$$\boldsymbol{\theta}^i_{t|t-1} = \boldsymbol{\theta}^i_{t-1|t-1}. \tag{S1.39}$$

The cross covariance is obtained as

$$\begin{aligned} \mathbf{W}^i_{t-1,t|t} &\equiv E_{\boldsymbol{\theta}^i_{t-1},\boldsymbol{\theta}^i_t|\mathbf{x}_{0:t}}\left(\boldsymbol{\theta}^i_{t-1} - \boldsymbol{\theta}^i_{t-1|t-1}\right)\left(\boldsymbol{\theta}^i_t - \boldsymbol{\theta}^i_{t|t-1}\right)^\top \\ &= E_{\boldsymbol{\theta}^i_{t-1},\boldsymbol{\xi}_t|\mathbf{x}_{0:t}}\left(\boldsymbol{\theta}^i_{t-1} - \boldsymbol{\theta}^i_{t-1|t-1}\right)\left(\boldsymbol{\theta}^i_{t-1} + \boldsymbol{\xi}_t - \boldsymbol{\theta}^i_{t-1|t-1}\right)^\top \\ &= \mathbf{W}^i_{t-1|t-1} + E_{\boldsymbol{\theta}^i_{t-1},\boldsymbol{\xi}_t|\mathbf{x}_{0:t}}\left[\left(\boldsymbol{\theta}^i_{t-1} - \boldsymbol{\theta}^i_{t-1|t-1}\right)\boldsymbol{\xi}_t^\top\right] \\ &= \mathbf{W}^i_{t-1|t-1}. \end{aligned} \tag{S1.40}$$

Here, at the second equality, we inserted the state equation with a state noise $\boldsymbol{\xi}_{t-1}$, and used $\boldsymbol{\theta}^i_{t|t-1} = \boldsymbol{\theta}^i_{t-1|t-1}$. The last equality is obtained due to the orthogonality of the fluctuation of $\boldsymbol{\theta}^i_{t-1}$ and noise $\boldsymbol{\xi}_t$.

Given the joint density, we obtain the conditional density (Eq. S1.35). We note that given the multivariate normal distribution,

$$\mathbf{x} = \begin{bmatrix} \mathbf{x}_a \\ \mathbf{x}_b \end{bmatrix} \sim \mathcal{N}\left(\begin{bmatrix} \boldsymbol{\mu}_a \\ \boldsymbol{\mu}_b \end{bmatrix}, \begin{bmatrix} \boldsymbol{\Sigma}_{aa} & \boldsymbol{\Sigma}_{ab} \\ \boldsymbol{\Sigma}_{ba} & \boldsymbol{\Sigma}_{bb} \end{bmatrix} \right). \tag{S1.41}$$

The conditional distribution of $\mathbf{x}_a|\mathbf{x}_b$ follows

$$\mathbf{x}_a|\mathbf{x}_b \sim \mathcal{N}(\boldsymbol{\mu}_{a|b}, \boldsymbol{\Sigma}_{a|b}) \tag{S1.42}$$

with

$$\boldsymbol{\mu}_{a|b} = \boldsymbol{\mu}_a + \boldsymbol{\Sigma}_{ab}\boldsymbol{\Sigma}_{bb}^{-1}(\mathbf{x}_b - \boldsymbol{\mu}_b), \tag{S1.43}$$

$$\boldsymbol{\Sigma}_{a|b} = \boldsymbol{\Sigma}_{aa} - \boldsymbol{\Sigma}_{ab}\boldsymbol{\Sigma}_{bb}^{-1}\boldsymbol{\Sigma}_{ba}. \tag{S1.44}$$

Applying this formula, we obtain

$$p(\boldsymbol{\theta}^i_{t-1}|\boldsymbol{\theta}^i_t, \mathbf{x}_{0:t-1}, \mathbf{w}) = \mathcal{N}\left(\boldsymbol{\theta}^i_{t-1}; \boldsymbol{\theta}^i_{t-1|t-1} + \mathbf{A}_{t-1}(\boldsymbol{\theta}^i_t - \boldsymbol{\theta}^i_{t-1|t-1}), \mathbf{W}^i_{t-1|t-1} - \mathbf{A}_{t-1}\mathbf{W}^i_{t-1|t-1}\right), \tag{S1.45}$$

where $\mathbf{A}_{t-1} = \mathbf{W}^i_{t-1|t-1}(\mathbf{W}^i_{t|t-1})^{-1}$.

Finally, the smoothing density at time t is obtained by multiplying the smoother density at time t and integrating out $\boldsymbol{\theta}^i_t$ according to Eq. S1.34. For this, we note that, given the following two normal distributions:

$$p(\mathbf{x}_a|\mathbf{x}_b) = \mathcal{N}(\mathbf{x}_a; \mathbf{A}\mathbf{x}_b + \mathbf{b}, \boldsymbol{\Sigma}_{a|b}), \tag{S1.46}$$

$$p(\mathbf{x}_b) = \mathcal{N}(\mathbf{x}_b; \boldsymbol{\mu}_b, \boldsymbol{\Sigma}_b), \tag{S1.47}$$

the marginal distribution of $\mathbf{x}_a$ is obtained as

$$p(\mathbf{x}_a) = \int p(\mathbf{x}_a|\mathbf{x}_b)\, p(\mathbf{x}_b)\, d\mathbf{x}_b = \mathcal{N}(\mathbf{x}_a; \boldsymbol{\mu}_a, \boldsymbol{\Sigma}_a), \tag{S1.48}$$

where

$$\boldsymbol{\mu}_a = \mathbf{A}\boldsymbol{\mu}_b + \mathbf{b}, \tag{S1.49}$$

$$\boldsymbol{\Sigma}_a = \mathbf{A}\boldsymbol{\Sigma}_b\mathbf{A}^\top + \boldsymbol{\Sigma}_{a|b}. \tag{S1.50}$$

Applying this formula to Eq. S1.45 and $p(\boldsymbol{\theta}^i_t|\mathbf{x}_{0:T}, \mathbf{w}) = \mathcal{N}\left(\boldsymbol{\theta}^i_t; \boldsymbol{\theta}^i_{t|T}, \mathbf{W}^i_{t|T}\right)$, we obtain the smoothing density $p(\boldsymbol{\theta}^i_{t-1}|\mathbf{x}_{0:T}, \mathbf{w})$ whose mean and covariance are given by

$$\boldsymbol{\theta}^i_{t-1|T} = \boldsymbol{\theta}^i_{t-1|t-1} + \mathbf{A}_{t-1}(\boldsymbol{\theta}^i_{t|T} - \boldsymbol{\theta}^i_{t-1|t-1}), \tag{S1.51}$$

and

$$\begin{aligned} \mathbf{W}^i_{t-1|T} &= \mathbf{A}_{t-1}\mathbf{W}^i_{t|T}\mathbf{A}^\top_{t-1} + \mathbf{W}^i_{t-1|t-1} - \mathbf{A}_{t-1}\mathbf{W}^i_{t-1|t-1} \\ &= \mathbf{W}^i_{t-1|t-1} + \mathbf{A}_{t-1}\mathbf{W}^i_{t|T}\mathbf{A}^\top_{t-1} - \mathbf{A}_{t-1}\mathbf{W}^i_{t|t-1}(\mathbf{W}^i_{t|t-1})^{-1}\mathbf{W}^i_{t-1|t-1} \\ &= \mathbf{W}^i_{t-1|t-1} + \mathbf{A}_{t-1}\left(\mathbf{W}^i_{t|T} - \mathbf{W}^i_{t|t-1}\right)\mathbf{A}^\top_{t-1}. \end{aligned} \tag{S1.52}$$

We thus obtained the backward recursion formulae to obtain the smoothing densities.

5. Optimization of hyperparameters

We consider the problem of optimizing the hyperparameters that maximize the marginal likelihood function. Instead of the marginal likelihood, we optimize its tractable lower bound. In the Expectation-Maximization (EM) algorithm, the posterior density is obtained under given hyperparameters via the algorithm described in the previous section

at the E-step. At the M-step, we optimize the hyperparameters that maximize the lower bound, using the given posterior density. Using Jensen's inequality $\log E[X] \geq E[\log X]$, this lower bound is given by

$$\begin{aligned} l(\mathbf{w}^*) &\equiv \log p(\mathbf{x}_{0:T}|\mathbf{w}^*) \\ &= \log \int p(\boldsymbol{\theta}_{1:T}|\mathbf{x}_{0:T},\mathbf{w}) \frac{p(\mathbf{x}_{0:T},\boldsymbol{\theta}_{1:T}|\mathbf{w}^*)}{p(\boldsymbol{\theta}_{1:T}|\mathbf{x}_{0:T},\mathbf{w})} d\boldsymbol{\theta}_{1:T} \\ &= \log E_{\boldsymbol{\theta}_{1:T}|\mathbf{x}_{0:T},\mathbf{w}} \frac{p(\mathbf{x}_{0:T},\boldsymbol{\theta}_{1:T}|\mathbf{w}^*)}{p(\boldsymbol{\theta}_{1:T}|\mathbf{x}_{0:T},\mathbf{w})} \\ &\geq E_{\boldsymbol{\theta}_{1:T}|\mathbf{x}_{0:T},\mathbf{w}} \log \frac{p(\mathbf{x}_{0:T},\boldsymbol{\theta}_{1:T}|\mathbf{w}^*)}{p(\boldsymbol{\theta}_{1:T}|\mathbf{x}_{0:T},\mathbf{w})} \\ &= E_{\boldsymbol{\theta}_{1:T}|\mathbf{x}_{0:T},\mathbf{w}} \log p(\mathbf{x}_{0:T},\boldsymbol{\theta}_{1:T}|\mathbf{w}^*) - E_{\boldsymbol{\theta}_{1:T}|\mathbf{w}} \log p(\boldsymbol{\theta}_{1:T}|\mathbf{x}_{0:T},\mathbf{w}). \end{aligned} \tag{S1.53}$$

The first term is called the Q-function:

$$\begin{aligned} \tilde{Q}(\mathbf{w}) &= E_{\boldsymbol{\theta}_{1:T}|\mathbf{x}_{0:T},\mathbf{w}} \log p(\mathbf{x}_{0:T},\boldsymbol{\theta}_{1:T}|\mathbf{Q}) \\ &= E_{\boldsymbol{\theta}_{1:T}|\mathbf{x}_{0:T},\mathbf{w}} \log p(\mathbf{x}_{0:T}|\boldsymbol{\theta}_{1:T},\mathbf{Q}) + E_{\boldsymbol{\theta}_{1:T}|\mathbf{x}_{0:T},\mathbf{w}} \log p(\boldsymbol{\theta}_{1:T}|\mathbf{Q}). \end{aligned} \tag{S1.54}$$

The second term is the entropy of the posterior density, which is fixed at M-step. We thus optimize the hyperparameters that maximize the Q-function. More explicitly, the Q-function can be written as

$$\begin{aligned} \tilde{Q}(\mathbf{w}) &= E_{\boldsymbol{\theta}_{1:T}|\mathbf{x}_{0:T},\mathbf{w}} \sum_{t=1}^{T}\sum_{i=1}^{N}\sum_{l=1}^{L} [(\boldsymbol{\theta}_t^i)^T \mathbf{F}(x_{i,t}^l, \mathbf{x}_{t-1}^l) - \psi(\mathbf{x}_{t-1}^l)] \\ &+ E_{\boldsymbol{\theta}_{1:T}|\mathbf{x}_{0:T},\mathbf{w}} \sum_{i=1}^{N} \left[-\frac{1}{2}\log|2\pi\boldsymbol{\Sigma}^i| - \frac{1}{2}(\boldsymbol{\theta}_1^i - \boldsymbol{\mu}^i)^\top (\boldsymbol{\Sigma}^i)^{-1} (\boldsymbol{\theta}_1^i - \boldsymbol{\mu}^i) \right] \\ &+ E_{\boldsymbol{\theta}_{1:T}|\mathbf{x}_{0:T},\mathbf{w}} \sum_{t=2}^{T}\sum_{i=1}^{N} \left[-\frac{1}{2}\log|2\pi\mathbf{Q}^i| - \frac{1}{2}(\boldsymbol{\theta}_t^i - \boldsymbol{\theta}_{t-1}^i)^\top (\mathbf{Q}^i)^{-1} (\boldsymbol{\theta}_t^i - \boldsymbol{\theta}_{t-1}^i) \right]. \end{aligned} \tag{S1.55}$$

Our objective is to choose $\mathbf{Q}$ such that the function $\tilde{Q}(\mathbf{Q})$ attains an extremum. By noting

$$\frac{\partial \log|2\pi\mathbf{Q}^i|}{\partial \mathbf{Q}^i} = \frac{1}{|\mathbf{Q}^i|}\frac{\partial|\mathbf{Q}^i|}{\partial \mathbf{Q}^i} = \frac{1}{|\mathbf{Q}^i|}|\mathbf{Q}^i|(\mathbf{Q}^i)^{-1} = (\mathbf{Q}^i)^{-1}, \tag{S1.56}$$

and

$$\begin{aligned} \frac{\partial}{\partial \mathbf{Q}^i}(\boldsymbol{\theta}_t^i - \boldsymbol{\theta}_{t-1}^i)^\top (\mathbf{Q}^i)^{-1} (\boldsymbol{\theta}_t^i - \boldsymbol{\theta}_{t-1}^i) &= \frac{\partial (\mathbf{Q}^i)^{-1}}{\partial \mathbf{Q}^i} \frac{\partial}{\partial (\mathbf{Q}^i)^{-1}} (\boldsymbol{\theta}_t^i - \boldsymbol{\theta}_{t-1}^i)^\top (\mathbf{Q}^i)^{-1} (\boldsymbol{\theta}_t^i - \boldsymbol{\theta}_{t-1}^i) \\ &= -(\mathbf{Q}^i)^{-2} (\boldsymbol{\theta}_t^i - \boldsymbol{\theta}_{t-1}^i)(\boldsymbol{\theta}_t^i - \boldsymbol{\theta}_{t-1}^i)^\top, \end{aligned} \tag{S1.57}$$

we obtain

$$\frac{\partial \tilde{Q}(\mathbf{w})}{\partial \mathbf{Q}^i} = E_{\boldsymbol{\theta}_{1:T}|\mathbf{x}_{1:T},\mathbf{w}} \sum_{t=2}^{T} \left[-\frac{1}{2}(\mathbf{Q}^i)^{-1} + \frac{1}{2}(\mathbf{Q}^i)^{-2}(\boldsymbol{\theta}_t^i - \boldsymbol{\theta}_{t-1}^i)(\boldsymbol{\theta}_t^i - \boldsymbol{\theta}_{t-1}^i)^\top \right]. \tag{S1.58}$$

Setting the above derivative equal to zero, it follows that the optimal $\mathbf{Q}^i$ is obtained as

$$\mathbf{Q}^i = \frac{1}{T-1}\sum_{t=2}^{T} E_{\boldsymbol{\theta}_{1:T}|\mathbf{x}_{1:T},\mathbf{w}} (\boldsymbol{\theta}_t^i - \boldsymbol{\theta}_{t-1}^i)(\boldsymbol{\theta}_t^i - \boldsymbol{\theta}_{t-1}^i)^\top. \tag{S1.59}$$

We note that the expectation in the above equation can be decomposed into

$$E_{\boldsymbol{\theta}_{1:T}|\mathbf{x}_{1:T},\mathbf{w}} \left[\boldsymbol{\theta}_t^i(\boldsymbol{\theta}_t^i)^\top - \boldsymbol{\theta}_{t-1}^i(\boldsymbol{\theta}_t^i)^\top - \boldsymbol{\theta}_t^i(\boldsymbol{\theta}_{t-1}^i)^\top + \boldsymbol{\theta}_{t-1}^i(\boldsymbol{\theta}_{t-1}^i)^\top \right]. \tag{S1.60}$$

Hence, using the following definitions of the equal-time covariance matrix:

$$\mathbf{W}_{t|T}^i = E_{\boldsymbol{\theta}_{1:T}|\mathbf{x}_{0:T},\mathbf{w}} \boldsymbol{\theta}_t^i(\boldsymbol{\theta}_t^i)^\top - \boldsymbol{\theta}_{t|T}^i(\boldsymbol{\theta}_{t|T}^i)^\top, \tag{S1.61}$$

and the delayed covariance:

$$\mathbf{W}^i_{t,t-1|T} = E_{\boldsymbol{\theta}_{1:T}|\mathbf{x}_{0:T},\mathbf{w}}(\boldsymbol{\theta}^i_t - \boldsymbol{\theta}^i_{t|T})(\boldsymbol{\theta}^i_{t-1} - \boldsymbol{\theta}^i_{t-1|T})^\top$$
$$= E_{\boldsymbol{\theta}_{1:T}|\mathbf{x}_{0:T},\mathbf{w}}\boldsymbol{\theta}^i_t(\boldsymbol{\theta}^i_{t-1})^\top - \boldsymbol{\theta}^i_{t|T}(\boldsymbol{\theta}^i_{t-1|T})^\top, \tag{S1.62}$$

the optimal $\mathbf{Q}^i$ is obtained as

$$\mathbf{Q}^i = \frac{1}{T-1}\sum_{t=2}^{T}\left[(\boldsymbol{\theta}^i_{t|T} - \boldsymbol{\theta}^i_{t-1|T})(\boldsymbol{\theta}^i_{t|T} - \boldsymbol{\theta}^i_{t-1|T})^\top + \mathbf{W}^i_{t|T} - \mathbf{W}^i_{t-1,t|T} - \mathbf{W}^i_{t,t-1|T} + \mathbf{W}^i_{t-1|T}\right], \tag{S1.63}$$

where $\mathbf{W}^i_{t-1,t|T} = (\mathbf{W}^i_{t,t-1|T})^\top$. We compute the lag-one smoothed covariance following the method of De Jong and Mackinnon [2]:

$$\mathbf{W}^i_{t,t-1|T} = \mathbf{W}^i_{t|t}(\mathbf{W}^i_{t+1|t})^{-1}\mathbf{W}^i_{t|T}. \tag{S1.64}$$

Similarly, we update $\boldsymbol{\Sigma}^i$ according to

$$\boldsymbol{\Sigma}^i = \mathbf{W}^i_{1|T} + (\boldsymbol{\theta}^i_{1|T} - \boldsymbol{\mu})(\boldsymbol{\theta}^i_{1|T} - \boldsymbol{\mu})^\top. \tag{S1.65}$$

6. Approximate log marginal likelihood function

The convergence of the EM algorithm was assessed using the log marginal likelihood. Below, we derive the approximate solution for the log marginal likelihood of the kinetic Ising model.

First, we note that the marginal likelihood function $p(\mathbf{x}_{0:T}|\mathbf{w})$ can be expressed as follows:

$$p(\mathbf{x}_{0:T}|\mathbf{w}) = p(\mathbf{x}_0)\prod_{t=1}^{T} p(\mathbf{x}_t|\mathbf{x}_{0:t-1},\mathbf{w})$$
$$= p(\mathbf{x}_0)\prod_{t=1}^{T}\int d\boldsymbol{\theta}_t p(\mathbf{x}_t|\mathbf{x}_{0:t-1},\boldsymbol{\theta}_t,\mathbf{w})p(\boldsymbol{\theta}_t|\mathbf{x}_{0:t-1},\mathbf{w})$$
$$= p(\mathbf{x}_0)\prod_{t=1}^{T}\int d\boldsymbol{\theta}_t p(\mathbf{x}_t|\mathbf{x}_{t-1},\boldsymbol{\theta}_t)p(\boldsymbol{\theta}_t|\mathbf{x}_{0:t-1},\mathbf{w})$$
$$= p(\mathbf{x}_0)\prod_{t=1}^{T}\prod_{i=1}^{N}\int d\boldsymbol{\theta}^i_t \prod_{l=1}^{L} p(x^l_{i,t}|\mathbf{x}_{t-1},\boldsymbol{\theta}^i_t)p(\boldsymbol{\theta}^i_t|\mathbf{x}_{0:t-1},\mathbf{w}). \tag{S1.66}$$

The observation model and the one-step prediction density in the equation above are written as

$$\prod_{l=1}^{L} p(x^l_{i,t}|\mathbf{x}^l_{t-1},\boldsymbol{\theta}^i_t) = \prod_{l=1}^{L}\exp\left[\theta_{i,t}x^l_{i,t} + \sum_{j=1}^{N}\theta_{ij,t}x^l_{it}x^l_{j,t-1} - \psi(\boldsymbol{\theta}^i_t,\mathbf{x}^l_{t-1})\right]$$
$$= \exp\left[(\boldsymbol{\theta}^i_t)^T\sum_{l=1}^{L}\mathbf{F}(x^l_{i,t},\mathbf{x}^l_{t-1}) - \sum_{l=1}^{L}\psi(\boldsymbol{\theta}^i_t,\mathbf{x}^l_{t-1})\right], \tag{S1.67}$$

and

$$p(\boldsymbol{\theta}^i_t|\mathbf{x}_{0:t-1},\mathbf{w}) = \frac{1}{\sqrt{|2\pi\mathbf{W}^i_{t|t-1}|}}\exp\left[-\frac{1}{2}(\boldsymbol{\theta}^i_t - \boldsymbol{\theta}^i_{t|t-1})^\top(\mathbf{W}^i_{t|t-1})^{-1}(\boldsymbol{\theta}^i_t - \boldsymbol{\theta}^i_{t|t-1})\right]. \tag{S1.68}$$

Substituting Eqs.S1.67 and S1.68 into Eq.S1.66, we obtain

$$p(\mathbf{x}_{0:T}|\mathbf{w}) = p(\mathbf{x}_0)\prod_{i=1}^{N}\prod_{t=1}^{T}\int d\boldsymbol{\theta}^i_t\frac{1}{\sqrt{|2\pi\mathbf{W}^i_{t|t-1}|}}$$
$$\cdot\exp\left[(\boldsymbol{\theta}^i_t)^T\sum_{l=1}^{L}\mathbf{F}(x^l_{i,t},\mathbf{x}^l_{t-1}) - \sum_{l=1}^{L}\psi(\boldsymbol{\theta}^i_t,\mathbf{x}^l_{t-1}) - \frac{1}{2}(\boldsymbol{\theta}^i_t - \boldsymbol{\theta}^i_{t|t-1})^\top(\mathbf{W}^i_{t|t-1})^{-1}(\boldsymbol{\theta}^i_t - \boldsymbol{\theta}^i_{t|t-1})\right]. \tag{S1.69}$$

We now define the function $q(\boldsymbol{\theta}_t^i)$ as follows:

$$q(\boldsymbol{\theta}_t^i) = (\boldsymbol{\theta}_t^i)^T \sum_{l=1}^{L} \mathbf{F}(x_{i,t}^l, \mathbf{x}_{t-1}^l) - \sum_{l=1}^{L} \psi(\boldsymbol{\theta}_t^i, \mathbf{x}_{t-1}^l) - \frac{1}{2}(\boldsymbol{\theta}_t^i - \boldsymbol{\theta}_{t|t-1}^i)^\top (\mathbf{W}_{t|t-1}^i)^{-1} (\boldsymbol{\theta}_t^i - \boldsymbol{\theta}_{t|t-1}^i). \tag{S1.70}$$

The Taylor expansion of $q(\boldsymbol{\theta}_t^i)$ around $\boldsymbol{\theta}^*$ up to the second order yields

$$q(\boldsymbol{\theta}_t^i) = q(\boldsymbol{\theta}^*) + \left.\frac{\partial q(\boldsymbol{\theta}_t^i)}{\partial \boldsymbol{\theta}_t^i}\right|_{\boldsymbol{\theta}_t^i = \boldsymbol{\theta}^*} (\boldsymbol{\theta}_t^i - \boldsymbol{\theta}^*) + \frac{1}{2}(\boldsymbol{\theta}_t^i - \boldsymbol{\theta}^*)^\top \left.\frac{\partial^2 q(\boldsymbol{\theta}^i)}{\partial \boldsymbol{\theta}_t^i \partial (\boldsymbol{\theta}_t^i)^\top}\right|_{\boldsymbol{\theta}_t^i = \boldsymbol{\theta}^*} (\boldsymbol{\theta}_t^i - \boldsymbol{\theta}^*). \tag{S1.71}$$

The value of $\boldsymbol{\theta}_t^i$ that maximizes the function $q(\boldsymbol{\theta}_t^i)$ is the MAP estimate $\boldsymbol{\theta}_{t|t}^i$ of the filter density. Further, the quadratic term evaluated at the MAP estimate is given by the negative inverse of the filter covariance $\mathbf{W}_{t|t}^i$. Hence, at $\boldsymbol{\theta}^* = \boldsymbol{\theta}_{t|t}^i$, the Taylor expansion becomes

$$q(\boldsymbol{\theta}_t^i) \simeq q(\boldsymbol{\theta}_{t|t}^i) - \frac{1}{2}(\boldsymbol{\theta}_t^i - \boldsymbol{\theta}_{t|t}^i)^\top (\mathbf{W}_{t|t}^i)^{-1} (\boldsymbol{\theta}_t^i - \boldsymbol{\theta}_{t|t}^i). \tag{S1.72}$$

With this quadratic approximation, the marginal likelihood is obtained as

$$\begin{aligned} p(\mathbf{x}_{0:T}|\mathbf{w}) &\simeq p(\mathbf{x}_0) \prod_{t=1}^{T} \prod_{i=1}^{N} \int d\boldsymbol{\theta}_t^i \frac{1}{\sqrt{|2\pi \mathbf{W}_{t|t-1}^i|}} \exp\left[q(\boldsymbol{\theta}_{t|t}^i) - (\boldsymbol{\theta}_t^i - \boldsymbol{\theta}_{t|t}^i)^\top \frac{1}{2} [\mathbf{W}_{t|t}^i]^{-1} (\boldsymbol{\theta}_t^i - \boldsymbol{\theta}_{t|t}^i) \right] \\ &= p(\mathbf{x}_0) \prod_{t=1}^{T} \prod_{i=1}^{N} \exp[q(\boldsymbol{\theta}_{t|t}^i)] \frac{\sqrt{|2\pi \mathbf{W}_{t|t}^i|}}{\sqrt{|2\pi \mathbf{W}_{t|t-1}^i|}} \frac{1}{\sqrt{|2\pi \mathbf{W}_{t|t}^i|}} \int d\boldsymbol{\theta}_t^i \exp\left[-(\boldsymbol{\theta}_t^i - \boldsymbol{\theta}_{t|t}^i)^\top \frac{1}{2} [\mathbf{W}_{t|t}^i]^{-1} (\boldsymbol{\theta}_t^i - \boldsymbol{\theta}_{t|t}^i) \right] \\ &= p(\mathbf{x}_0) \prod_{t=1}^{T} \prod_{i=1}^{N} \sqrt{\frac{|2\pi \mathbf{W}_{t|t}^i|}{|2\pi \mathbf{W}_{t|t-1}^i|}} \exp[q(\boldsymbol{\theta}_{t|t}^i)]. \end{aligned} \tag{S1.73}$$

We thus obtain the log marginal likelihood function as follows:

$$\log p(\mathbf{x}_{0:T}|\mathbf{w}) \simeq \log p(\mathbf{x}_0) + \sum_{t=1}^{T} \sum_{i=1}^{N} \left[\frac{1}{2} \log |\mathbf{W}_{t|t}^i| - \frac{1}{2} \log |\mathbf{W}_{t|t-1}^i| + q(\boldsymbol{\theta}_{t|t}^i) \right]. \tag{S1.74}$$

Supplementary Note 2: An alternative calculation of the backward conditional entropy

Here, we give an alternative approach to obtaining the backward conditional entropy to the one given in Methods. The result gives an identical approximate solution.

Under the approximation of the following probabilities by independent distributions:

$$p(\mathbf{x}_{t-2}) = Q(\mathbf{x}_{t-2}), \tag{S2.1}$$
$$p(\mathbf{x}_t|\mathbf{x}_{t-1}) = Q(\mathbf{x}_t), \tag{S2.2}$$

the backward conditional entropy is approximated as

$$\begin{aligned}\sigma_t^{\mathrm{backward}} &= -\sum_{\mathbf{x}_{t-2}}\sum_{\mathbf{x}_{t-1}} p(\mathbf{x}_{t-1}|\mathbf{x}_{t-2})p(\mathbf{x}_{t-2})\sum_{\mathbf{x}_t} p(\mathbf{x}_t|\mathbf{x}_{t-1})\sum_i \left[x_{i,t-1}h_{i,t}(\mathbf{x}_t) - \psi(h_{i,t}(\mathbf{x}_t))\right] \\ &\simeq -\sum_{\mathbf{x}_{t-2}}\sum_{\mathbf{x}_{t-1}} p(\mathbf{x}_{t-1}|\mathbf{x}_{t-2})Q(\mathbf{x}_{t-2})\sum_{\mathbf{x}_t} Q(\mathbf{x}_t)\sum_i \left[x_{i,t-1}h_{i,t}(\mathbf{x}_t) - \psi(h_{i,t}(\mathbf{x}_t))\right] \\ &= -\sum_i\sum_{x_{i,t-1}}\sum_{\mathbf{x}_{t-2}} p(x_{i,t-1}|\mathbf{x}_{t-2})Q(\mathbf{x}_{t-2})\sum_{\mathbf{x}_t} Q(\mathbf{x}_t)\left[x_{i,t-1}h_{i,t}(\mathbf{x}_t) - \psi(h_{i,t}(\mathbf{x}_t))\right].\end{aligned} \tag{S2.3}$$

Let us define $\tilde{\phi}_{i,t}(x_{i,t})$ as

$$\tilde{\phi}_{i,t}(x_{i,t-1}) = \sum_{\mathbf{x}_t} Q(\mathbf{x}_t)\left[x_{i,t-1}h_{i,t}(\mathbf{x}_t) - \psi(h_{i,t}(\mathbf{x}_t))\right]. \tag{S2.4}$$

Using

$$\gamma(h_{i,t}) = x_{i,t-1}h_{i,t} - \psi(h_{i,t}), \tag{S2.5}$$

we approximate $\tilde{\phi}_{i,t}(x_{i,t})$ as

$$\tilde{\phi}_{i,t}(x_{i,t-1}) \approx \int \mathcal{D}_z\, \gamma(g_{i,t} + z\sqrt{\Delta_{i,t}}), \tag{S2.6}$$

where $\mathcal{D}_z = \frac{\mathrm{d}z}{\sqrt{2\pi}}\exp\left(-\frac{1}{2}z^2\right)$.

Then, the backward conditional entropy is written as

$$\begin{aligned}\sigma_t^{\mathrm{backward}} &= -\sum_{\mathbf{x}_{t-2}}\sum_{\mathbf{x}_{t-1}} p(\mathbf{x}_{t-1}|\mathbf{x}_{t-2})Q(\mathbf{x}_{t-2})\tilde{\phi}_{i,t}(x_{i,t-1}) \\ &= -\sum_i\sum_{x_{i,t-1}}\left(\sum_{\mathbf{x}_{t-2}} p(x_{i,t-1}|\mathbf{x}_{t-2})Q(\mathbf{x}_{t-2})\right)\tilde{\phi}_{i,t}(x_{i,t-1}).\end{aligned} \tag{S2.7}$$

Note that, from Eq. 52, we have

$$\begin{aligned} m_{i,t} &= \sum_{\mathbf{x}_{t-1}} p(\mathbf{x}_t = 1|\mathbf{x}_{t-1})p(\mathbf{x}_{t-1}) \simeq \sum_{\mathbf{x}_{t-1}} p(\mathbf{x}_t = 1|\mathbf{x}_{t-1})Q(\mathbf{x}_{t-1}), \\ 1 - m_{i,t} &= \sum_{\mathbf{x}_{t-1}} p(\mathbf{x}_t = 0|\mathbf{x}_{t-1})p(\mathbf{x}_{t-1}) \simeq \sum_{\mathbf{x}_{t-1}} p(\mathbf{x}_t = 0|\mathbf{x}_{t-1})Q(\mathbf{x}_{t-1}).\end{aligned} \tag{S2.8}$$

Applying these equations for the case of $t-1$, we obtain

$$\sigma_t^{\mathrm{backward}} \simeq -\sum_i \left\{m_{i,t-1}\tilde{\phi}_{i,t}(x_{i,t-1} = 1) + (1 - m_{i,t-1})\tilde{\phi}_{i,t}(x_{i,t-1} = 0)\right\}. \tag{S2.9}$$

Thus, it can be obtained by computing the two Gaussian integral terms.

Since this equation can be further computed as

$$\sigma_t^{\mathrm{backward}} \simeq -\sum_i \left\{m_{i,t-1}(\tilde{\phi}_{i,t}(x_{i,t-1} = 1) - \tilde{\phi}_{i,t}(x_{i,t-1} = 0)) + \tilde{\phi}_{i,t}(x_{i,t-1} = 0)\right\}. \tag{S2.10}$$

and

$$\tilde{\phi}_{i,t}(x_{i,t-1}=1)-\tilde{\phi}_{i,t}(x_{i,t-1}=0)=\sum_{\mathbf{x}_t} Q\left(\mathbf{x}_t\right) h_{i,t}(\mathbf{x}_t),$$
$$\tilde{\phi}_{i,t}(x_{i,t-1}=0)=-\sum_{\mathbf{x}_t} Q\left(\mathbf{x}_t\right) \psi(h_{i,t}(\mathbf{x}_t)), \tag{S2.11}$$

it becomes

$$\sigma_t^{\text{backward}}=-\sum_i\sum_{\mathbf{x}_t} Q(\mathbf{x}_t)\left[m_{i,t-1}h_{i,t}(\mathbf{x}_t)-\psi(h_{i,t}(\mathbf{x}_t))\right], \tag{S2.12}$$

which is equivalent to Eq. 57 in Methods and can be also approximated by the Gaussian integral.

Supplementary Note 3: Mean-field entropy flow under specific conditions

In this section, we derive the mean-field approximation of the entropy flow under the steady-state conditions or for independent neurons.

First, let us summarize the mean-field entropy flow. It is obtained as

$$\begin{aligned}\sigma_t^{\text{flow}} &= -\sigma_t^{\text{forward}} + \sigma_t^{\text{backward}} \\ &\approx \sum_i \int \mathcal{D}_z \left[-\chi\left(g_{i,t,t-1} + z\sqrt{\Delta_{i,t,t-1}}\right) + \phi_{i,t}\left(g_{i,t,t} + z\sqrt{\Delta_{i,t,t}}\right)\right], \end{aligned} \tag{S3.1}$$

where $g_{i,t,s}$ and $\Delta_{i,t,s}$ $(s = t, t-1)$ are given as

$$g_{i,t,s} = \theta_{i,t} + \sum_j \theta_{ij,t} m_{j,s}, \tag{S3.2}$$

$$\Delta_{i,t,s} = \sum_j \theta_{ij,t}^2 m_{j,s}(1 - m_{j,s}). \tag{S3.3}$$

Here $m_{j,s}$ is the mean-field activation rate of the j-th neuron at time s.

Using $r(h) = 1/(1 + e^{-h})$ and $\psi(h) = -\log(1 - r(h))$, $\chi(h)$ and $\phi_{i,t}(h)$ are given as

$$\begin{aligned}\chi(h) &= -r(h)\log r(h) - (1 - r(h))\log(1 - r(h)) \\ &= -r(h)\log\frac{r(h)}{1 - r(h)} - \log(1 - r(h)) \\ &= -r(h)h + \psi(h), \end{aligned} \tag{S3.4}$$

and

$$\phi_{i,t}(h) = -m_{i,t-1}h + \psi(h). \tag{S3.5}$$

1. Steady-state solution

Under the steady-state assumption ($m_{i,t} = m_{i,t-1} \equiv m_i$), we have $g_{i,t,t-1} = g_{i,t,t} \equiv g_i$ and $\Delta_{i,t,t-1} = \Delta_{i,t,t} \equiv \Delta_i$, making the inputs to χ and $\phi_{i,t}$ common for each neuron. Then, using Eqs. S3.4 and S3.5 with the common $h = g_i + z\sqrt{\Delta_i}$, we have

$$\begin{aligned}\sigma_t^{\text{flow}} &\approx \sum_i \int \mathcal{D}_z \left(r\left(g_i + z\sqrt{\Delta_i}\right) - m_i\right)\cdot\left(g_i + z\sqrt{\Delta_i}\right) \\ &= \sum_i \int \mathcal{D}_z \left(r\left(g_i + z\sqrt{\Delta_i}\right) - m_i\right)\cdot z\sqrt{\Delta_i}. \end{aligned} \tag{S3.6}$$

The term $r\left(g_i + z\sqrt{\Delta_i}\right) - m_i$ represents how the neuron's activity rate deviates from its long-term average, while $z\sqrt{\Delta_i}$ is the fluctuating input to that neuron. Thus, the mean-field solution for the steady state provides an intuitive picture of entropy flow as a measure of the neuron's causal response to fluctuations in its input.

The non-negativity of the mean-field entropy flow can be formally confirmed by Stein's lemma $E(f(X)(X - \mu)) = \sigma^2 E(f'(X))$ for a Gaussian random variable X with expectation μ and variance σ^2. By identifying $f(h) = r(h) - m_i$, $h - g_i = z\sqrt{\Delta_i}$, and $f'(h) = r'(h)$, it can be written as

$$\sigma_t^{\text{flow}} \approx \sum_i \Delta_i \left(\int \mathcal{D}_z\, r'(g_i + z\sqrt{\Delta_i})\right), \tag{S3.7}$$

where $r'(h) = r(h)(1 - r(h))$. Since $\Delta_i \geq 0$ and $r'(h) \geq 0$, the entropy flow is non-negative, which satisfies the requested property of the entropy flow at the steady state. However, while insightful, this form also reveals a key limitation of the approximation: the zero entropy flow is realized only at $\theta_{ij} = 0$ (except for $r = 0, 1$). Consequently, it does not correctly reduce to zero for symmetric couplings, failing to fully incorporate the distinction between symmetric and asymmetric interactions.

2. Independent neurons

Here we consider independent neurons (i.e., no couplings $\theta_{ij} = 0$) with time-varying field $\theta_{i,t}$. The entropy flow in this system is caused solely by the time-varying fields, or equivalently, the activity rate of individual neurons.

In this case, we have

$$g_{i,t,s} = \theta_{i,t}, \tag{S3.8}$$

$$\Delta_{i,t,s} = 0, \tag{S3.9}$$

which is independent of s, making the inputs to χ and $\phi_{i,t}$ common once again. Then, we have

$$\begin{aligned}\sigma_t^{\text{flow}} &\approx \sum_i \left(r\left(\theta_{i,t}\right) - m_{i,t-1}\right) \cdot \theta_{i,t} \\ &= \sum_i \left(m_{i,t} - m_{i,t-1}\right) \cdot \theta_{i,t}.\end{aligned} \tag{S3.10}$$

For $\theta_{i,t} < 0$, which corresponds to $m_{i,t} < 0.5$, a decrease in the activity rate $m_{i,t} - m_{i,t-1} < 0$ yields positive entropy flow, and an increase in the activity rate induces negative entropy flow.

Supplementary Note 4: The d-prime measure

Here, we provide the definition of the primary behavioral metric, d' (*d-prime*), for clarity. This follows the white paper of "Allen Brain Observatory: Visual Behavior Neuropixels", where further details are available.

To evaluate the sensitivity of the mice to the stimulus, the primary behavioral metric, d', was calculated using data detected only in the active condition with visual changes. The formula for d' is as follows:

$$d' = Z(R_H) - Z(R_F), \tag{S4.1}$$

where R_H is the hit rate (the proportion of trials in which the mouse correctly responded to a change in the visual stimulus), and R_F is the false alarm rate (the proportion of trials in which the mouse incorrectly responded to a non-existent change). The function Z represents the inverse of the cumulative distribution function of a standard normal distribution, converting the hit and false alarm rates into z-scores. To prevent extreme values (e.g., 0 or 1) from distorting the results, R_H and R_F were adjusted using the following boundary equations:

$$\frac{1}{2N_H} \leq R_H \leq 1 - \frac{1}{2N_H}, \quad \frac{1}{2N_F} \leq R_F \leq 1 - \frac{1}{2N_F}, \tag{S4.2}$$

where N_H and N_F are the total number of trials for the hit and false alarm conditions, respectively. To assess the overall behavioral performance across sessions or experimental conditions, mean d' was used as an aggregated measure, representing the average d' over multiple trials or sessions. For more details, see [3].

Supplementary Note 5: Entropy flow of high-firing neurons and behavioral performance

To elucidate how individual neurons increase total entropy flow in the active condition despite a smaller fraction of neurons exhibiting substantial firing rates (Supplementary Fig. S2, Fig. S3, and Fig. S4), we examined the relationship between the entropy flow and spike rates of individual neurons.

As shown in Eqs. 14 and 15, the mean-field entropy flow can be decomposed into contributions from individual neurons. We computed the entropy flow of individual neurons under the active and passive conditions and compared them with their firing rates (Supplementary Fig. S8**A**, mouse 574078). The dotted lines connect the values for the active (red) and passive (blue) conditions. We then investigated whether the change in the entropy flow by the behavioral conditions depends on the neuron's firing rate. Supplementary Fig. S8**B** shows the relationship between the geometric mean spike rates of the two conditions (abscissa) and the difference in entropy flow (ordinate) for individual neurons. The difference was computed as 'active' - 'passive,' indicating that the positive value marks a larger entropy flow in the active condition. The positive Spearman rank correlation coefficient ($\rho = 0.22$) for this exemplary mouse suggests that neurons with higher spike rates contributed to increasing total entropy flow in the active condition, despite the summed entropy flow differences across all individual neurons being negative (-3.8331 for this mouse). However, significant variations in the rank correlations were observed across mice.

Assuming that fewer high-firing neurons in the sparsely active populations in the active condition play a critical role in sensory processing (i.e., sparse coding [4–6]) and that such sensory processing involves time-asymmetric causal patterns, we hypothesized that the above relationship between the spike rates and entropy flow change might be related to mice's cognitive performance. To evaluate the task sensitivity of the mice, we used the primary behavioral metric, d' (*mean d-prime*, see Supplementary Note 4 for its definition). The scatter plot in the left panel of Supplementary Fig. S8**C** illustrates the relationship between behavioral measures (mean d-prime) and the rank correlation of entropy flow change with spike rates for all mice for image 'im036_r'. The plot suggests a positive dependency between these two values ($\rho = 0.3578$ measured by the Spearman rank correlation). To confirm this result, we conducted the permutation test that compared the observed rank correlation of the scatter plot with those of the surrogate data constructed by permuting the values of mean d-prime (Supplementary Fig. S8**C** Right). The result confirms the statistical significance of the positive correlation ($p = 0.0304$).

To corroborate that the result does not reflect estimation error in couplings, we analyzed trial-shuffled data, which showed no clear trend (Supplementary Fig. S8**D**). A permutation test confirmed that the observed correlation yielded a non-significant p-value of 0.5063. This result confirms that the association between higher entropy flow and higher firing neurons in more task-sensitive mice was driven by significant changes in the coupling strengths between the active and passive conditions, rather than firing rate shifts or noise couplings.

However, the additional analyses on the images im012_r and im115_r revealed that these relations were not significantly correlated ('im012_r': $p = 0.574$; 'im115_r': $p = 0.333$, permutation test). Similar analysis replacing the difference of the entropy flow between active and passive conditions with the difference of the entropy flow per activity rate between active and passive conditions yielded non-significant results for these three images.

[1] Rauch, H. E., Tung, F. & Striebel, C. T. Maximum likelihood estimates of linear dynamic systems. *AIAA journal* **3**, 1445–1450 (1965).
[2] Jong, P. D. & Mackinnon, M. J. Covariances for smoothed estimates in state space models. *Biometrika* **75**, 601–602 (1988).
[3] Hautus, M. J., Macmillan, N. A. & Creelman, C. D. *Detection theory: A user's guide* (Routledge, 2021).
[4] Olshausen, B. A. & Field, D. J. Emergence of simple-cell receptive field properties by learning a sparse code for natural images. *Nature* **381**, 607–609 (1996).
[5] Olshausen, B. A. & Field, D. J. Sparse coding with an overcomplete basis set: A strategy employed by v1? *Vision research* **37**, 3311–3325 (1997).
[6] Foldiak, P. Sparse coding in the primate cortex. *The handbook of brain theory and neural networks* 895–898 (2003).

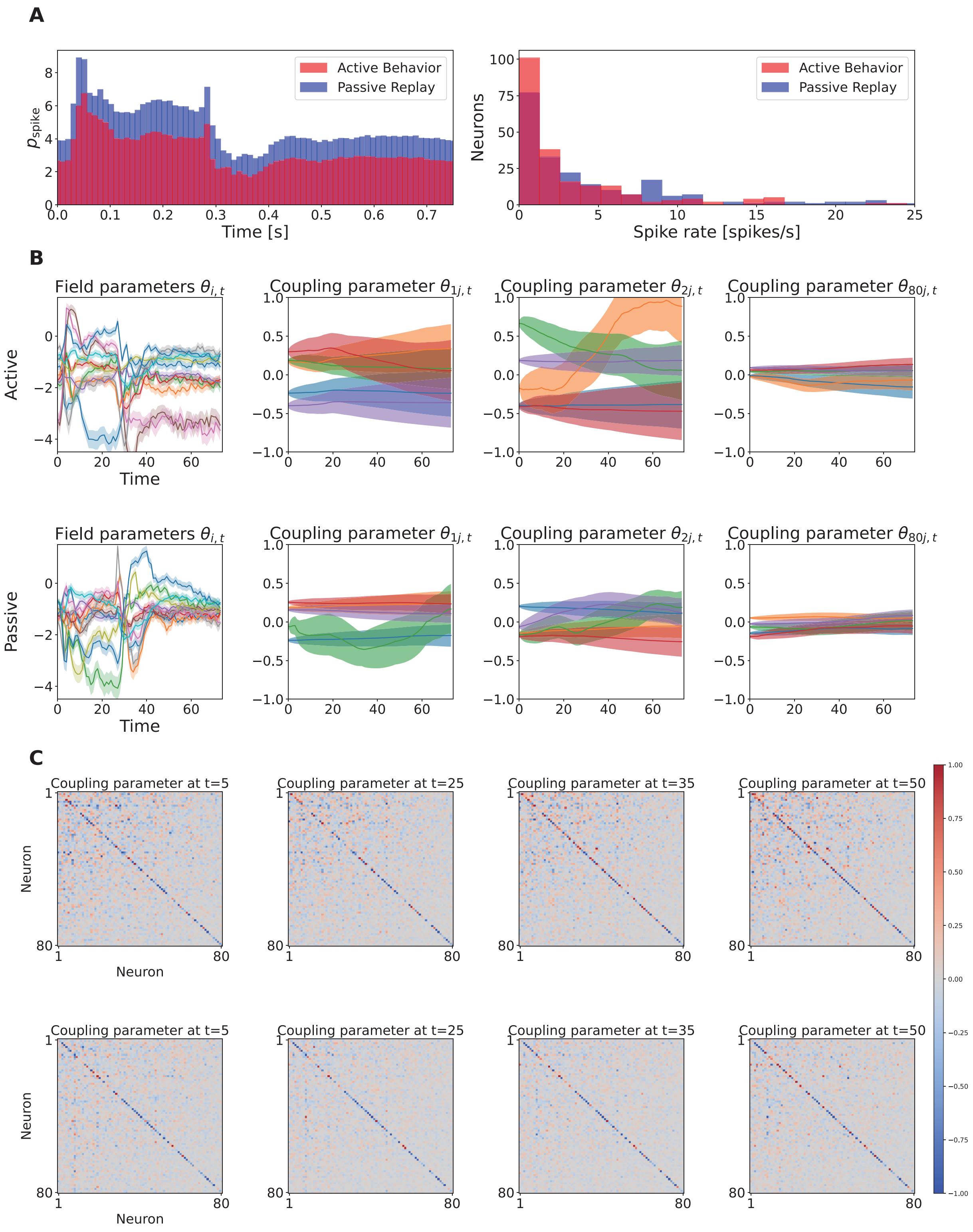

Supplementary Fig. S1. **Estimated neural dynamics under active and passive conditions in shuffled data of mouse 574078.** Presentation style follows Fig. 6.

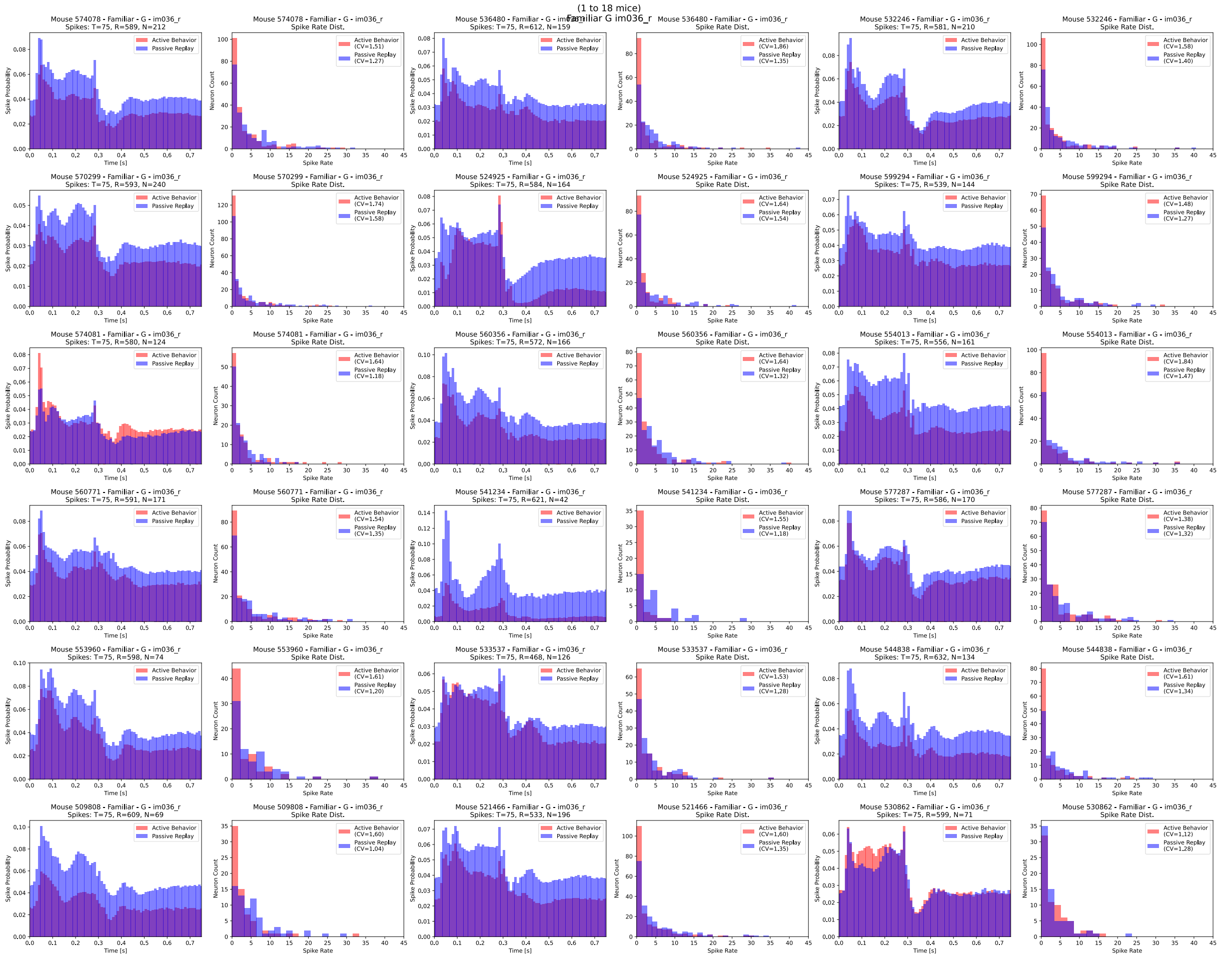

Supplementary Fig. S2. **Spike-rate dynamics and distributions for mice 1-18.** Spike-rate dynamics and distributions under the active (red) and passive (blue) conditions. The presentation styles for each mouse follow Fig. 6**A**. The mice were listed in descending order of behavioral performance measured by d-prime. See Supplementary Fig. S3 for the remaining mice.

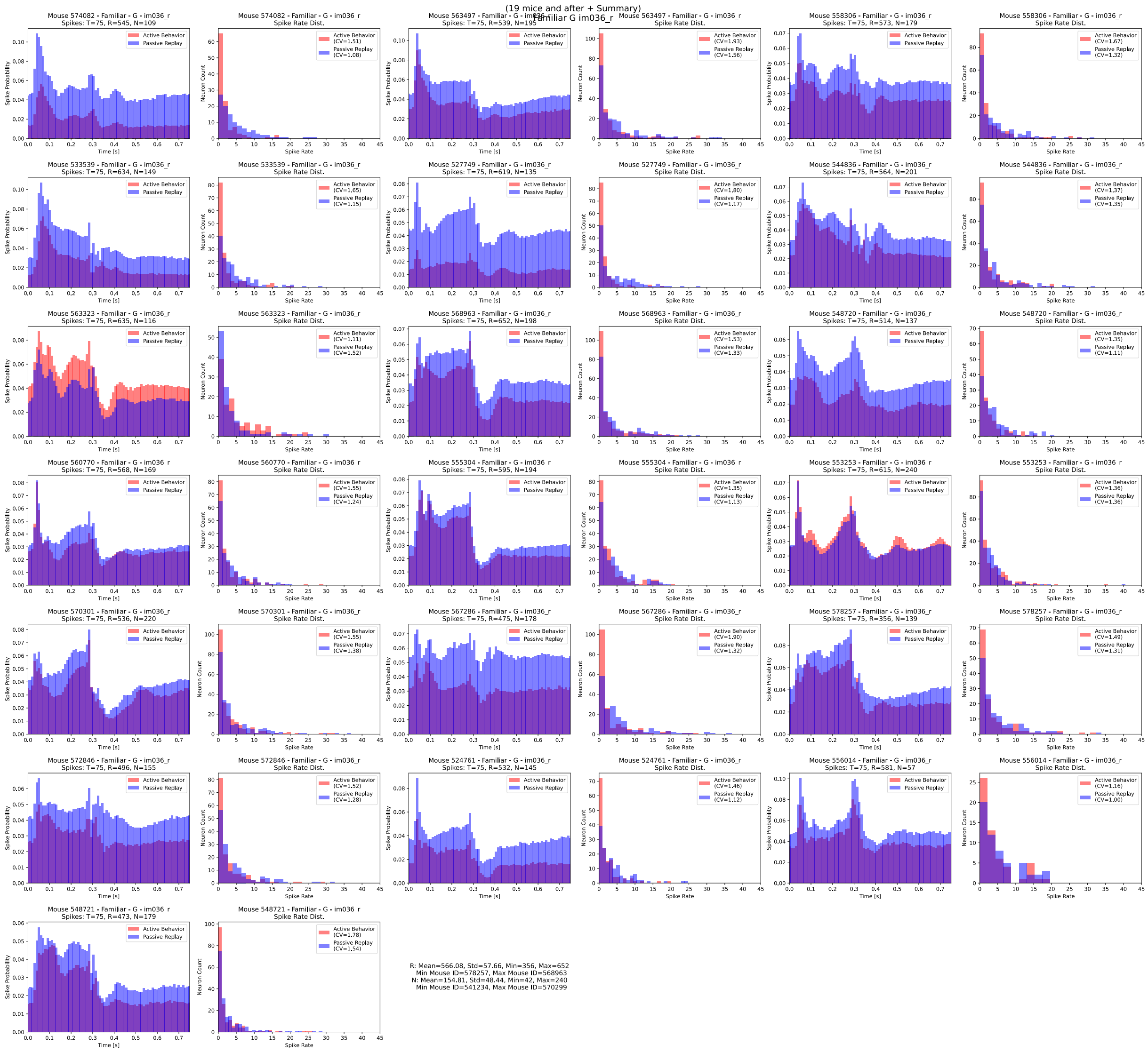

Supplementary Fig. S3. **Spike-rate dynamics and distributions for mice 19-37.** The same as in Supplementary Fig. S2 but for the remaining 19 mice.

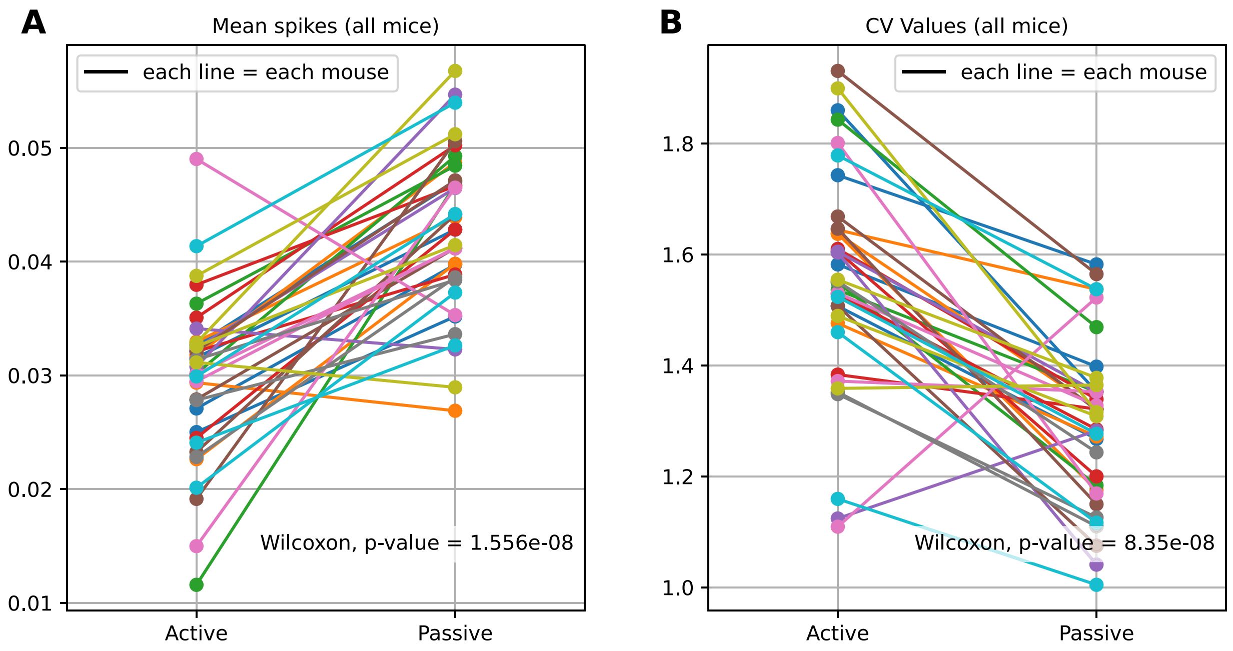

Supplementary Fig. S4. **Comparison of mean spiking probability and coefficient of variation in the active and passive conditions. A** Mean spiking probability across all bins, trials, and neurons in active and passive conditions. Each line represents the same mouse. Neurons showed significantly lower firing rates in the active condition ($p = 1.556 \times 10^{-8}$, Wilcoxon signed-rank test). **B** Coefficient of variations (CVs) of the firing rate distributions, a measure of sparseness, in the active and passive conditions. CV was significantly higher in the active condition ($p = 8.35 \times 10^{-8}$, Wilcoxon signed-rank test).

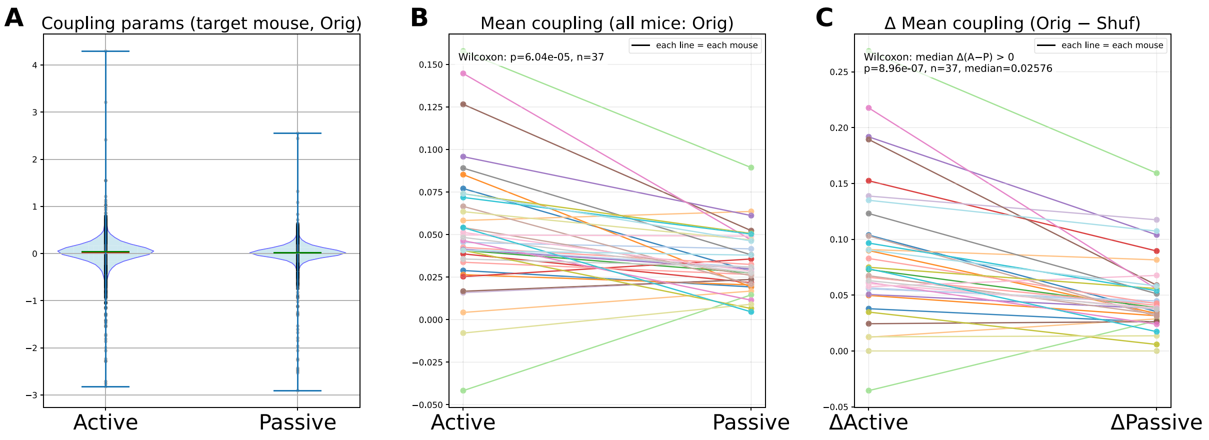

Supplementary Fig. S5. **Mean effective coupling and shuffle control.** **A** Violin plots of time-averaged effective couplings for mouse 574078 under the active and passive conditions. Horizontal bars indicate the mean (red) and median (green); points show individual entries. **B** Population summary of the per-mouse mean coupling in the original data; each line connects the active and passive values from the same mouse. Panel annotations report p-values and sample size (n) from Wilcoxon signed-rank tests across mice (two-sided). **C** Shuffle-adjusted means, where for each mouse the value in each condition is computed as (Original − Shuffle); lines connect paired values. The annotation reports a one-sided Wilcoxon signed-rank test assessing whether the median of {(Original − Shuffle) in Active} minus {(Original − Shuffle) in Passive} is greater than zero. Together, the results indicate that the mean effective coupling is larger in the active condition than in the passive condition, and that this increase persists after shuffle correction.

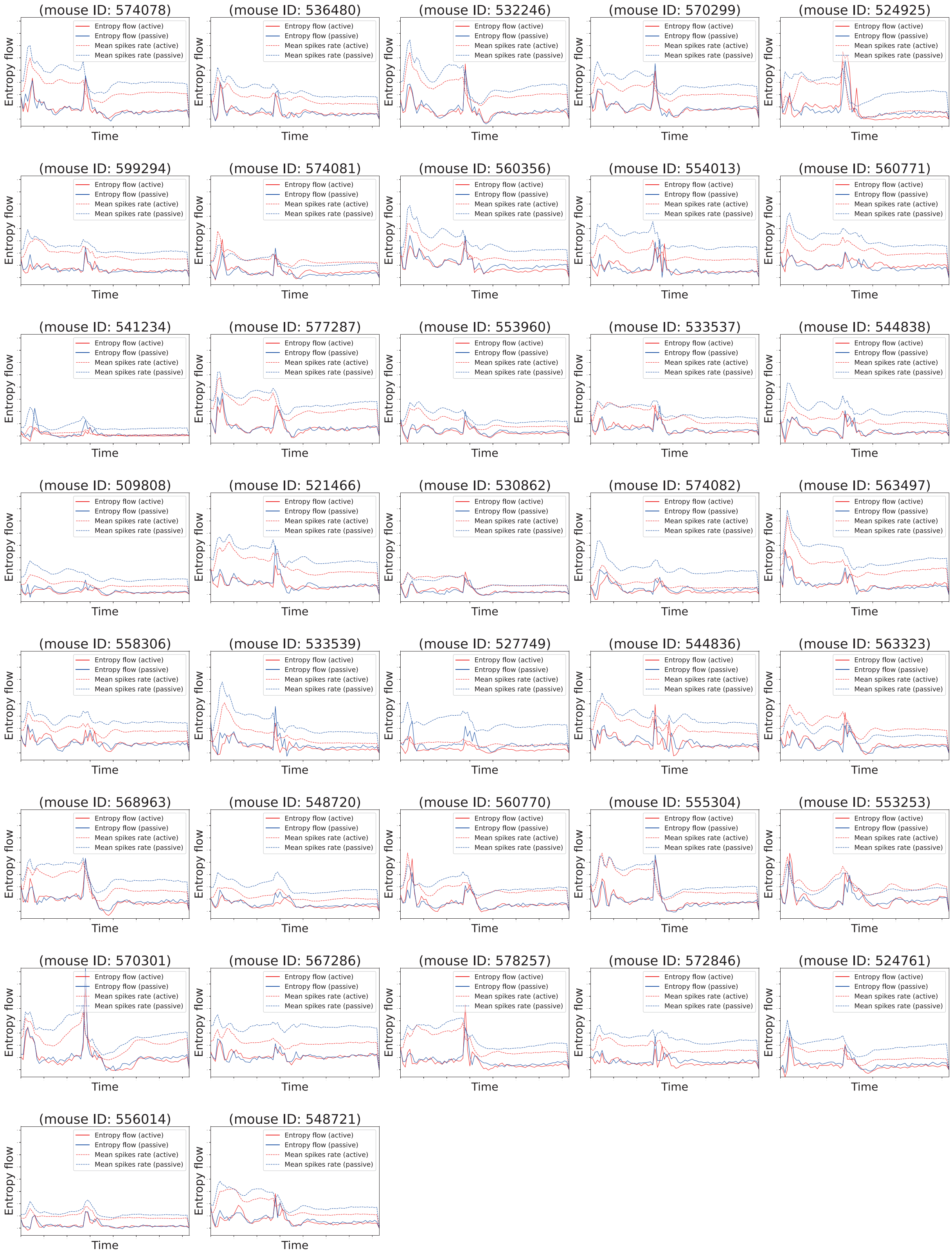

Supplementary Fig. S6. **Time courses of entropy flow and mean spike rates for each mouse under active and passive conditions.** Each subplot represents the dynamics of an individual mouse. Solid lines are entropy flows (red for active, blue for passive) while dashed lines represent the average population spike rate (red for active, blue for passive).

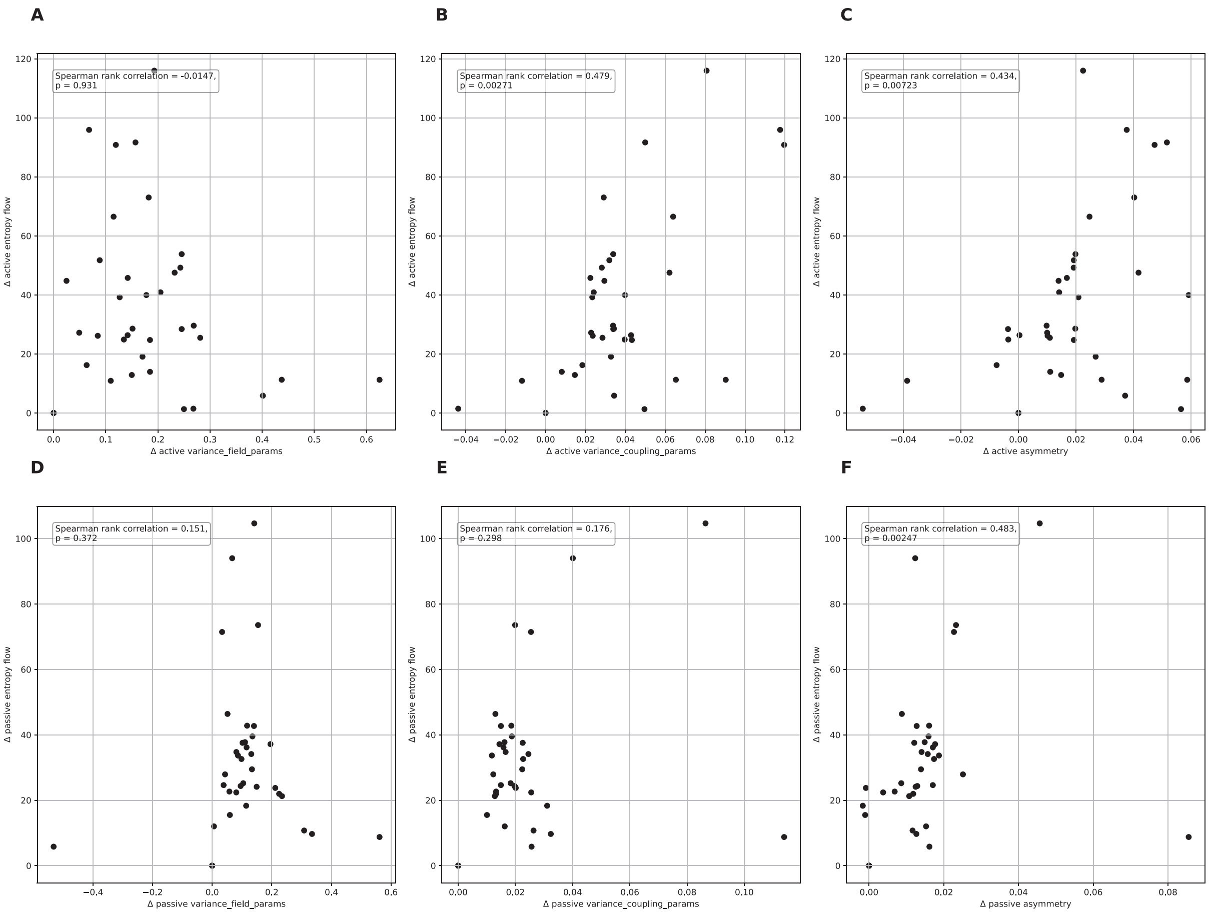

Supplementary Fig. S7. **Comparison of shuffle-subtracted parameter variabilities and coupling asymmetry with entropy flow for all mice.** Each row represents comparisons of parameter variabilities and coupling asymmetry (calculated by subtracting the shuffled-data estimate of the variance from the original-data estimate) and their relationship to the shuffle-subtracted entropy flow. **A, B, C** "Δactive" (shuffle-subtracted changes in the field, coupling variabilities, and coupling asymmetry) versus the shuffle-subtracted entropy flow in the active state. **D, E, F** "Δpassive" versus the shuffle-subtracted entropy flow in the passive state.

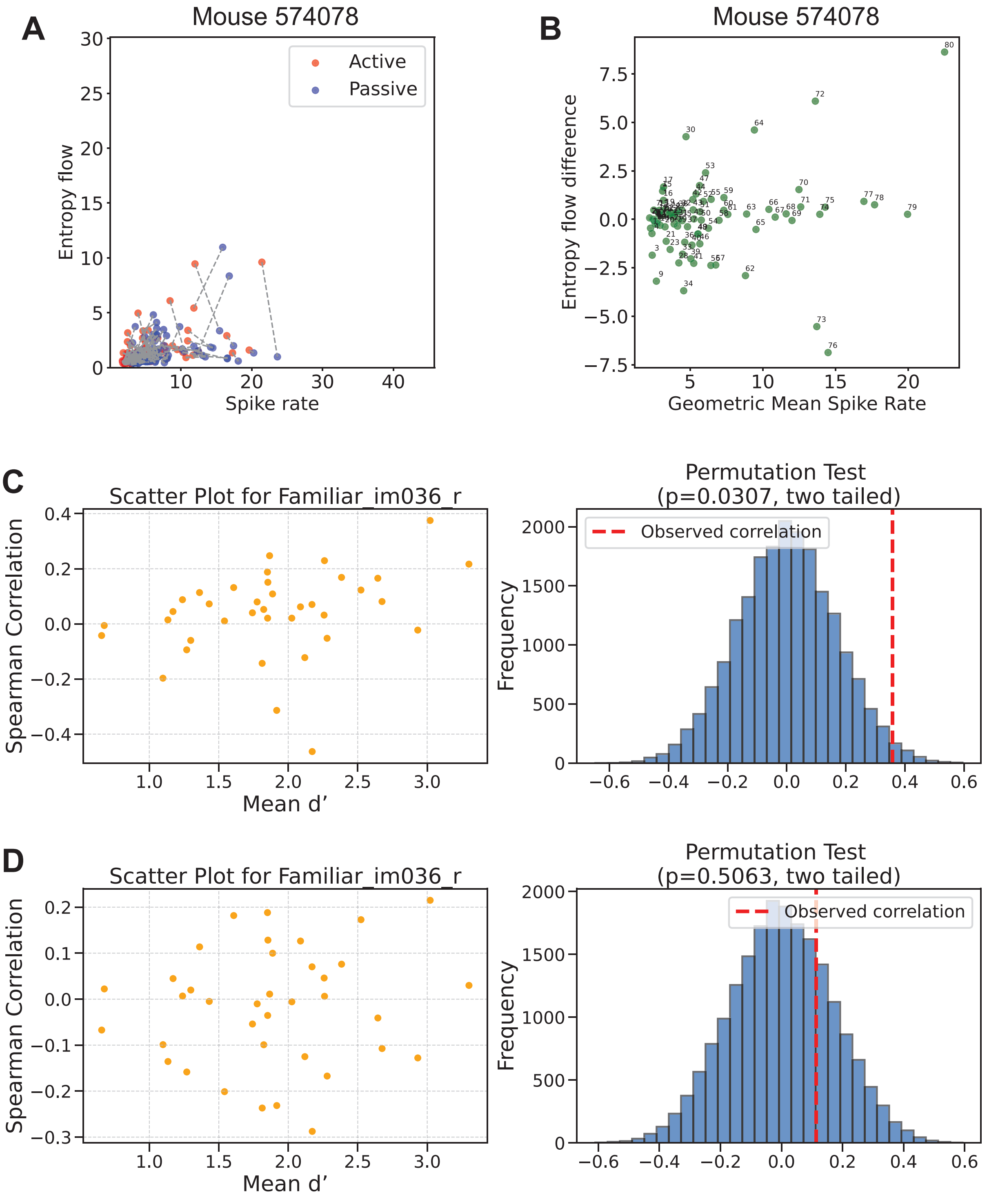

Supplementary Fig. S8. **Relating the dependency of entropy flow change of individual units on firing rates with behavioral performance.** **A** Mean spike rate vs entropy flow per individual unit under the active and passive conditions (mouse 574078). Dashed lines connect values for the two conditions, highlighting behavioral state-dependent changes. **B** Geometric mean spike rate (abscissa) vs differences in entropy flow (active - passive, ordinate) for individual units. The positive Spearman correlation coefficient ($\rho = 0.22$) suggests that units with higher spike rates increased entropy flow in the active condition. **C** (Left) Scatter plot of behavioral performance (mean d-prime) vs. the Spearman rank correlation between the geometric mean rate and entropy flow change of individual units. Each dot represents a single mouse. The dependency in this scatter plot was assessed again by the Spearman rank correlation coefficient, yielding $\rho = 0.3578$. (Right) A permutation test comparing the observed correlation value ρ with those obtained from the surrogate data. A statistically significant positive relationship was observed ($p = 0.0304$, two-tailed). The surrogate data was constructed by permuting the values of mean d-prime. **D** Results for trial-shuffled data.
